# Strain dependent differences in glucocorticoid-induced bone loss between C57BL/6J and CD-1 mice

**DOI:** 10.1038/srep36513

**Published:** 2016-11-04

**Authors:** Adel Ersek, Ana I. Espirito Santo, Youridies Vattakuzhi, Saumya George, Andrew R. Clark, Nicole J. Horwood

**Affiliations:** 1 The Kennedy Institute of Rheumatology, University of Oxford, Roosevelt Drive, Oxford, OX3 7FY, UK; 2 University of Birmingham, Edgbaston, Birmingham, B15 2TT, UK

## Abstract

We have investigated the effect of long-term glucocorticoid (GC) administration on bone turnover in two frequently used mouse strains; C57BL/6J and CD1, in order to assess the influence of their genetic background on GC-induced osteoporosis (GIO). GIO was induced in 12 weeks old female C57BL/6J and CD1 mice by subcutaneous insertion of long-term release prednisolone or placebo pellets. Biomechanical properties as assessed by three point bent testing revealed that femoral elasticity and strength significantly decreased in CD1 mice receiving GC, whereas C57BL/6J mice showed no differences between placebo and prednisolone treatment. Bone turnover assessed by microcomputer tomography revealed that contrary to C57BL/6J mice, prednisolone treated CD1 mice developed osteoporosis. *In vitro* experiments have underlined that, at a cellular level, C57BL/6J mice osteoclasts and osteoblasts were less responsive to GC treatment and tolerated higher doses than CD1 cells. Whilst administration of long-term release prednisolone pellets provided a robust GIO animal model in 12 weeks old CD1 mice, age matched C57BL/6J mice were not susceptible to the bone changes associated with GIO. This study indicates that for the induction of experimental GIO, the mouse strain choice together with other factors such as age should be carefully evaluated.

Glucocorticoids (GC) are steroid hormones secreted by the adrenal cortex that are involved in metabolic, developmental and immunological regulation. In the past years synthetic GC were often used as anti-inflammatory agents in the treatment of rheumatoid arthritis (RA)^1^.

To research the onset and treatment options of different chronic inflammatory diseases several mouse models that carry different mutations or lack the activity of certain genes have been established^2^,^3^,^4^. These animal models were and are very useful in revealing the involvement of different pathways in specific inflammatory diseases. Nonetheless, in humans different treatment regimens involving long-term GC administration usually leads to osteopenia, a side effect that was also observed in Swiss mice and related strains such as the CD1 mice^5^,^6^,^7^,^8^. However, other relevant mouse strains used for chronic inflammatory diseases that are regularly subjected to GC treatment in their research regimen have been established on the C57BL/6J background^2^,^9^. Recently Thiele and *et al*., have shown that long-term GC treatment in C57BL/6 animal model does not result in significant bone loss as opposed to its effects in humans or in other glucocorticoid-induced osteoporosis (GIO) susceptible mouse strains. Thus, in an effort to obtain some subtle osteopenia they have recommended to substantially increase the GC dose in the C57BL/6 mice in comparison to other mouse strains^10^.

To date, the C57BL/6 strain is frequently used to generate transgenic and knockout mice used in RA and other inflammatory research, but the data on the GC treatment side effects especially GIO in these mice remains very poor with only a few studies and all of them using older mice^7^,^11^. Therefore, in an effort to determine the effects of long term GC treatment *in vivo* and *in vitro* we have compared 12 weeks old (skeletally mature) non-GIO susceptible C57BL/6J strain with 12 weeks old CD1 mouse (Swiss-Webster type mouse) commonly used for GC induced osteopenia studies. Further, we have evaluated the strain specific responses to experimentally induced osteoporosis in the two main bone cell populations that are actively involved in bone remodelling.

Overall, our results have shown that the two strains exibit different profiles of bone turnover markers and biomechanical properties when subjected to long term GC administration and different sensitivity to GCs at a cellular level. Whilst 12 weeks old CD1 mice proved to be a robust GIO animal model with bone cells highly sensitive to low doses of GC, C57BL/6J mice were much less susceptible to GIO and bone cells from this strain were far less sensitive to GC exposure.

## Materials and Methods

### Animals and experimental procedures

12 weeks old female C57BL/6J and CD1 mice (Charles River UK Ltd. Margate, UK) were maintained in Biological Services Unit, The Kennedy Institute of Rheumatology, University of Oxford (Oxford, UK). Animal care and experimental procedures were approved by the University of Oxford Local Ethical Review Committee under PPL 70-7335 and were conducted in accordance with the UK Home Office Animals Act of 1986. GIO was induced in C57BL/6J and CD1 mice by subcutaneously implanted slow release prednisolone (2.5 mg/60 days) or placebo pellets (Innovative Research of America, USA). Mice were sacrificed 56 days after pellet insertion.

### *In vitro* osteoclast (OC) formation and tartrate resistant acid phosphatase (TRAP) assay

Bone marrow (BM) cells isolated from 12 weeks old C57BL/6J and CD1 mice were density separated with Lympholyte-M (Cedarlane, Burlington, USA) and the purified lymphocytes were cultured overnight at 37 °C in 5% CO_2_ in complete medium: α-MEM with 10% foetal bovine serum (FBS), supplemented with 50 ng/ml murine macrophage colony stimulating factor (M-CSF) (Miltenyi, Abingdon, UK) and next day were plated at a density of 1 × 10^5 cells/well in 96-well plates where OC precursors were induced to proliferate and differentiate with (150 ng/ml) M-CSF and (10–100 ng/ml) receptor activator of NF-kB ligand (RANKL) (Miltenyi, Abingdon, UK) supplemented with 0–10^4 nM Dexamethasone (Dex) (Sigma-Aldrich, Gillingham, UK) as indicated. On day 6 cells were washed with PBS and fixed with 4% paraformaldehyde (PFA), then permeabilized with acetone/ethanol 1:1 solution and stained for TRAP activity for 15 mins at 37 °C (0.1 mg/ml Napthol AS-MX phosphate, 0.4 mg/ml Fast Red Violet LB Salt, 0.5 ml Dimethylformamide in 50 ml TRAP buffer; all from Sigma-Aldrich, Haverhill Suffolk, UK). Multinucleated (no. of nuclei ≥3) TRAP-positive cells that stained red were considered to be OCs. In all the experiments BM cells were also cultured in complete medium with M-CSF only served as negative control for OC formation.

### *In vitro* osteogenensis assay

Murine calvarial osteoblasts (OBL) isolated as described previously^12^ at day 1 of life by sequential enzymatic digestion with 1 mg/ml Collagenase II (Worthington, Lakewood, USA) and 2 mg/ml Dispase (Invitrogen, Paisley, UK) in PBS^12^ were plated directly in 96 well plates at 5 × 10^4 cells/well in control medium (DMEM and 10% FBS) or osteogenic medium (DMEM supplemented with 10% FBS, 1 M β-glycerolphosphate, 50 mg/ml phosphoascorbic acid and 0–10^4 nM Dex). For evaluating mineralized calvarial bone nodules on day 6 of culture, cells were stained with 1% alizarin red-S (Sigma-Aldrich, Gillingham, UK). The alizarin red-positive area was analysed using NIH image software Image-J, version 1.45d (imagej.nih.gov/ij/) and expressed as a percentage of alizarin red-positive area over total area of the well^13^,^14^. In all the experiments OBL were also cultured in control medium as negative control for mineralization.

### Microcomputer tomography (Micro-CT) analysis

Mouse lumbar vertebrae and tibia were harvested 56 days after pellet insertion were fixed in 4% PFA and scanned with Skyscan 1174 scanner (SkyScan, Belgium), 50 kV, 800 μA, 8.3 μm isometric voxel resolution, 0.7 degree rotation step with 0.5 mm Al filter and exposure set to 7008 ms and at 12.57 μm image pixel size. Images were analysed using Skyscan CT Analyzer software version 1.13.2.1.

### Decalcified bone histology and histomorphometric analysis

Mouse vertebrae were fixed in 4% PFA at 4 °C. After 24 h of fixation, vertebrae were transferred into 10% EDTA for 21 days while changing the EDTA solution every 3–4 days. After paraffin embedding following the regular techniques, the bones were sectioned with Sakura Accu-Cut SRM 200 microtome (Japan). Sections (4 μm) were stained for Masson’s trichrome or TRAP activity and counterstained with 0.25% methyl green following the standard procedures. Immunohistochemistry for osteocalcin was performed following trypsin antigen retrieval then primary antibody overnight at 4 °C (Osteocalcin mouse polyclonal antibody produced in rabbit, ALX-210-333-C100, Enzo, used at final conc. 1 mg/ml). For non-specific control staining treat sections with isotype control antibody at a matched concentration (Rabbit IgG, polyclonal-Isotype Control, ab27478). A biotinylated secondary antibody (Biotinylated Goat anti-rabbit, ab64256, Abcam) was used for antibody detection before counterstaining with Mayers hematoxylin. Slides were viewed using Olympus Bx51 microscope (Japan) and pictures were taken with Olympus DP71 camera (Japan).

Histomorphometric analysis of bone volume, bone surface and determination of OC numbers and OC surface were carried out using the OsteoMeasure histomorphometry system (OsteoMetrics Inc. Atlanta, GA, USA) in accordance with ASBMR standard nomenclature on TRAP stained and methyl green counterstained histological sections as described^15^,^16^.

### Three-point bend testing

Three-point bend testing was performed using an Instron 5942 materials-testing load frame and custom-built mounts that incorporated rounded supports to minimise cutting and shear loads (Instron® Limited, USA). The right femur of mice treated with placebo or prednisolone were cleaned of soft tissues and fixed in 70% ethanol at 4 °C. Prior to bend testing bones were rehydrated in PBS for two hours. Femurs were placed horizontally and centred on two support points and positioned with the anterior surface upward. Load was applied vertically downwards to the midshaft with a constant rate of displacement of 2 mm/sec until failure. Based on the load-deformation curve, the maximum load and elastic modulus was determined. The load-deformation curves were plotted using Bluehill 3 software (Instron® Limited, USA).

### Enzyme linked immunosorbent assay (ELISA)

RatLaps EIA kit for determining serum carboxy-terminal telopeptide of type I collagen (CTX) was obtained from IDS (Immunodiagnostics Systems Ltd., Boldon, UK). ELISA Kit for murine Procollagen I N-terminal Propeptide (PINP) was obtained from Caltag Medsystems (UK). ELISA assays were performed according to the manufacturer’s instructions.

### Statistical analysis

Statistical interpretation of the data was done using one-way analysis of variance (ANOVA) with a subsequent Tukey post-test determining the differences between the specific experimental groups. Data is represented as mean ± standard deviation (SD) or standard error of mean (SEM), as specified in figure legends. Statistical significance was set for a *p* value less than 0.05. All calculations were performed using GraphPad Prism version 5 software (GraphPad Prism, San Diego, USA).

## Results

Experimental GIO models are well established in outbred Swiss Webster mice or other strains such as CD1 developed on Swiss Webster background, in which a loss of bone volume and trabecular connectivity is observed^7^,^8^,^17^. However, most genetically modified mice are established on the C57BL/6 background. To investigate whether C57BL/6 mice are less responsive to GIO, slow release pellets containing prednisolone (3.2 mg/kg/day) were implanted in 12 weeks old female C57BL/6J and CD1 mice for 56 days.

### Biomechanical properties and microstructural characteristics in GC treated CD1 and C57BL/6 mice bones

**Figure 1:**
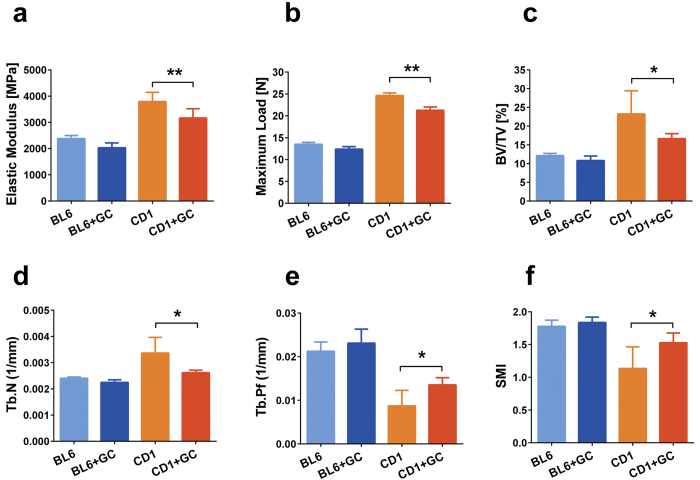
Bone biomechanical properties and structure analysis in C57BL/6J and CD1 mice treated with prednisolone or placebo. Femurs of female mice treated with placebo or 3.2 mg/kg/day prednisolone (GC) were harvested after 56 days and three-point bend testing was performed to test (**a**) elastic modulus and (**b**) maximum load. Lumbar vertebrae were analysed by microCT and (**c**) trabecular bone volume (BV/TV), (**d**) trabecular number, (**e**) trabecular pattern factor and (**f**) structure model index (SMI) were evaluated (n = 5 mice/group) in case of C57BL/6J (BL6) and CD1 mice. Error bars correspond to SD. Statistical analysis was performed using one-way ANOVA and Tukey’s multiple comparison test evaluated differences between groups (*p < 0.05, **p < 0.001).

**Figure 2:**
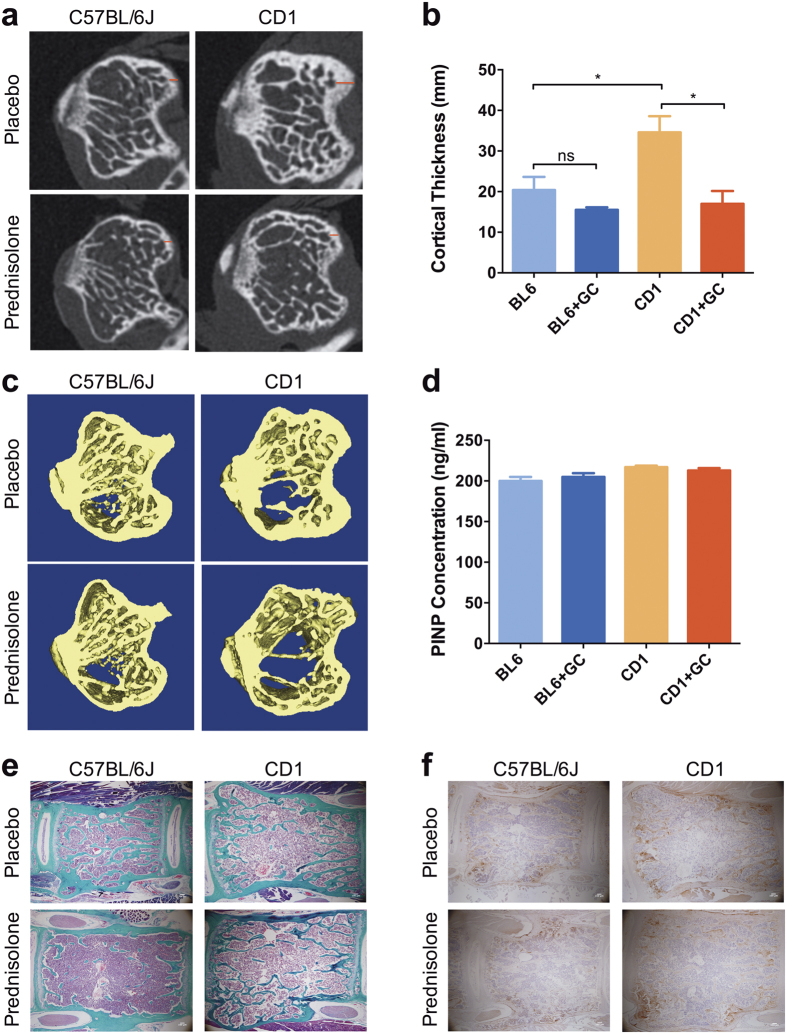
(**a**) Original unsegmented image of mouse proximal tibia cross-section placebo and prednisolone treated C57Bl/6J and CD1 mice. (**b**) 2D measurement of cortical thickness of placebo and prednisolone treated C57Bl/6J and CD1 mice. Error bars correspond to SEM. Statistical analysis was performed using unpaired t-test (*p < 0.05). (**c**) 3D micro-CT reconstruction of mouse proximal tibia cross-section placebo and prednisolone treated C57Bl/6J and CD1 mice. (**d**) PINP levels in serum samples of C57BL/6J and CD1 mice treated with either prednisolone or placebo. Error bars correspond to SEM. (**e**) Representative histological sections of mouse lumbar vertebrae stained for Masson Trichrome (magnification 4x). (**f**) Representative histological sections of mouse lumbar vertebrae stained for osteocalcin (magnification 4x).

Bone strength and elasticity were evaluated by three-point bend testing of long bones. Femurs obtained from prednisolone treated CD1 mice had significantly lower elasticity and were able to withstand a significantly lesser load compared to the placebo treated mice, while C57BL/6 mice femurs biomechanical properties were not affected by GC treatment (Fig. 1a,b). Vertebral bone architectural parameters evaluated by micro-CT revealed that the prednisolone treatment in CD1 mice significantly decreased trabecular bone volume (BV/TV) and trabecular number (Tb.N), while the trabecular pattern factor (Tb.Pf) and structure model index (SMI) increased significantly. Nonetheless, prednisolone treatment in C57BL/6 mice had no significant effect on vertebral trabecular parameters (Fig. 1c–f). This can be clearly visualised by 2D and 3D images of the tibia from these mice where the decrease in trabecular structure is seen in the CD1 mice yet the C57BL/6 mice have preserved trabecular parameters following GC treatment (Fig. 2a,c).

Examination of the cortical bone thickness likewise showed that there was a significant loss of bone in the CD1 mice following prednisolone treatment whereas the C57BL/6 mice did not lose cortical bone in response to treatment with GC (Fig. 2a,b). The loss in both cortical and trabecular thickness in the CD1 mice would account for the reduction in bend strength seen in Fig. 1a,b.

### *In vivo* osteoblast parameters were unchanged between CD1 and C57BL/6 mice

To assess the effects of prednisolone treatment on osteoblast parameters *in vivo* we collected serum from the mice at sacrifice and assessed the levels of murine procollagen I N-terminal propeptide (PINP) as a measure of osteoblast activity. There was no significant difference between the CD1 mice and C57BL/6 mice either with or without GC treatment (Fig. 2d). The bone architecture of the vertebrae was investigated by Masson’s trichrome staining and the distribution of osteoblasts was visualized by immunohistochemistry for osteocalcin, a mature osteoblast protein (Fig. 2e,f). There were no observable differences between the two different mouse strains therefore the effects of osteoclasts (OC) were next investigated.

### Osteoclastogenesis and OC activity in Prednisolone treated CD1 and C57BL/6 mice *in vivo*

**Figure 3:**
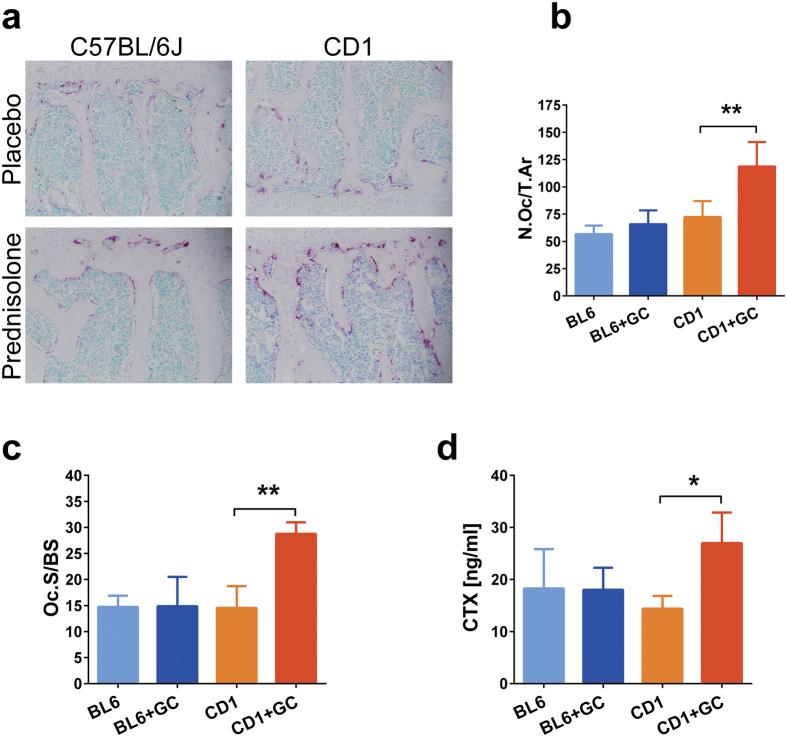
Bone histology and histomorphometry in placebo or prednisolone treated C57BL/6J and CD1 mice. Representative histological sections of mouse lumbar vertebrae (**a**) stained for OC TRAP activity and counterstained with 0.25% methyl green solution (magnification 20x). (**b,c**) Bone histomorphometry parameters obtained by analysing the histological sections of lumbar vertebrae from mice (n = 5 mice/group) from C57BL/6J (BL6) and CD1 mice treated with placebo or prednisolone (GC); Number of osteoclasts/total bone area (N.Oc/T.Ar); OC surface is expressed as percent of total bone surface (Oc.S/BS). (**c**) Serum values of CTX (ng/ml). Error bars correspond to SD. Statistical analysis was performed using one-way ANOVA and Tukey’s multiple comparison test evaluated differences between groups (*p < 0.05, **p < 0.01).

Histological analysis of the vertebrae performed after TRAP staining that identified OCs by red staining showed significantly increased OC numbers and surface area in the prednisolone treated CD1 mice compared to the placebo group, but no significant differences were observed in the case of C57BL/6 mice (Fig. 3a–c). To determine OC resorptive activity and bone turnover *in vivo*, serum was taken by cardiac puncture from prednisolone or placebo treated mice at the end of experiment and analysed for C-terminal telopeptides of collagen type I (CTX) by ELISA. CTX levels showed a significant increase in response to long-term prednisolone administration in CD1 mice, but no effect was observed in the case of C57BL/6 mice (Fig. 3d).

### Effect of Dexamethasone on *in vitro* osteoclastogenesis in CD1 and C57BL/6 mice cells

**Figure 4:**
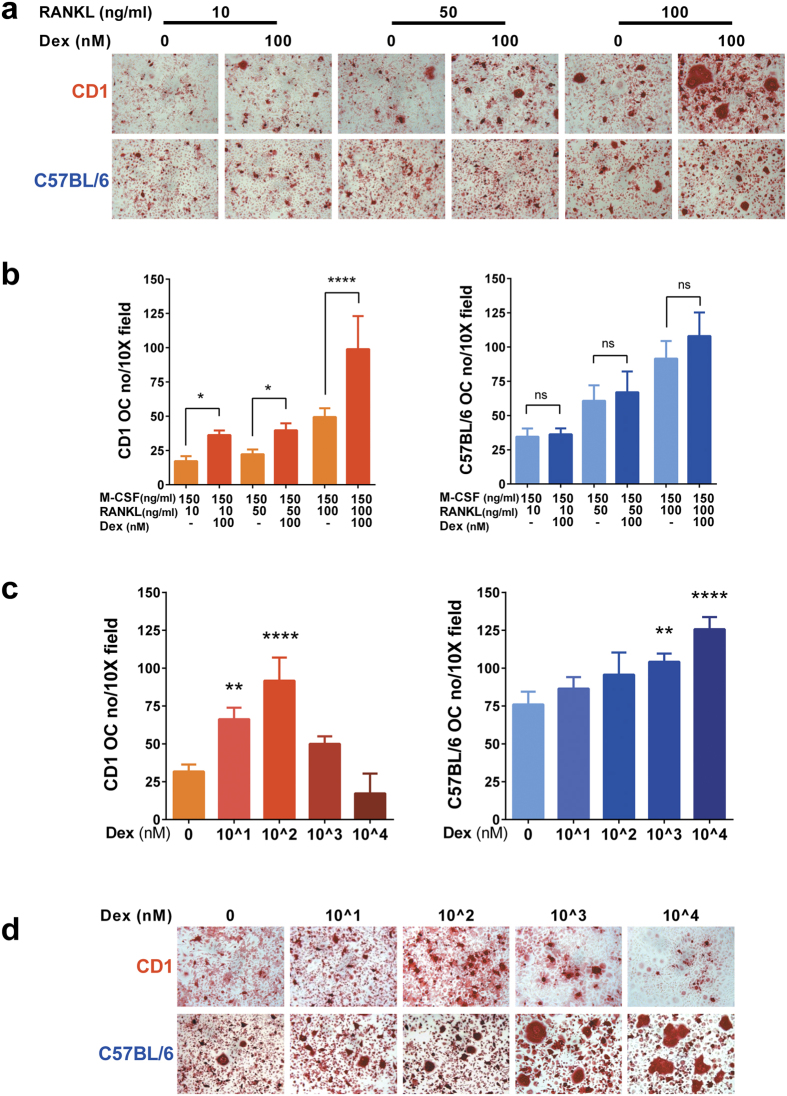
*In vitro* OC differentiation of C57BL/6J and CD1 cells in the presence of Dex. Overnight cultured (with 50 ng/ml M-CSF) C57BL/6J and CD-1 mice BM cells were plated in 96-well plates and treated with 150 ng/ml M-CSF and increasing doses of RANKL (10, 50, 100 ng/ml) in the presence or absence of 100 nM Dex. (Pictures in upper row were obtained from CD1 mice and pictures in lower row were obtained from C57BL/6 mice) (**a**) and TRAP positive multinucleated cells per 10X field were enumerated (**b**). Overnight cultured murine cells were induced to form OC with 150 ng/ml M-CSF and 100 ng/ml RANKL and with increasing concentrations of Dex (0, 10^1, 10^2, 10^3 and 10^4). (Pictures in upper row were obtained from CD1 mice and pictures in lower row were obtained from C57BL/6 mice) (**d**) and TRAP positive multinucleated cells per 10X field were counted (**c**). All pictures were taken with 20X magnification. A representative experiment of 3 (n = 3), each performed in triplicate assays is shown (**a**, **b**, **c** and **d**). Error bars correspond to SD. Statistical analysis was performed using one-way ANOVA followed by Tukey’s multiple comparison test (*p < 0.05, **p < 0.005, ****p < 0.0001).

In order to analyse the effect of GC on the main cellular components of the bone we induced BM cells from CD1 and C57BL/6 mice to form OCs *in vitro* by using M-CSF and different doses of RANKL (10, 50, 100 ng/ml) in the presence or absence of 100 nM Dex. In the absence of RANKL no OC were formed from either mouse strain BM (data not shown) and RANKL stimulated osteoclastogenesis dose dependently in both mouse strains (Fig. 4a,b). In the presence of RANKL, the addition of 100 nM Dex significantly increased OC numbers in the CD1 cultures, but not in the C57BL/6 BM cultures (Fig. 4a,b). To assess how different doses of Dex might influence osteoclastogenesis in CD1 and C57BL6 cells, overnight cultured murine BM cells (with 50 ng/ml M-CSF) were induced to form OCs with 150 ng/ml M-CSF and 100 ng/ml RANKL (the doses with the highest osteogenic potential) and with increasing concentrations of Dex (0, 10^1, 10^2, 10^3 and 10^4 nM). In CD1 mice the addition of Dex at low and medium doses (10^1 and 10^2 nM) significantly stimulated osteoclastogenesis while high doses of Dex (10^3, 10^4 nM) inhibited OC formation compared to the 10^2 nM dose (Fig. 4c,d). In case of C57BL/6 mice OC numbers and OC size were increased significantly only at higher doses (10^3, 10^4 nM) of Dex (Fig. 4c,d).

### Effect of Dexamethasone on *in vitro* bone nodule formation in CD1 and C57BL/6 mice cells

**Figure 5:**
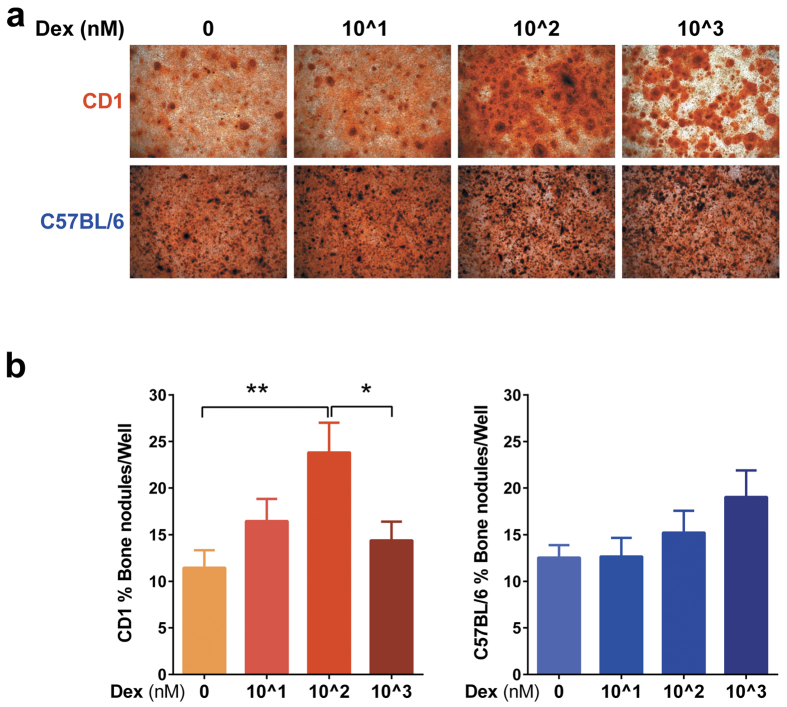
*In vitro* osteogenesis of C57BL/6J and CD1 mouse calvarial cells in the presence of Dex. Calvarial OBL were isolated from 1 day old pups by collagenase II + Dispase enzymatic digestion and were plated in 96 well plates in osteogenic induction media with different Dex concentrations (0, 10^1, 10^2 and 10^3 nM). OBL mineralization was evaluated on day 6 by 1% alizarin red-S staining (Original magnification, x4, Pictures in upper row were obtained from CD1 mice and pictures in lower row were obtained from C57BL/6 mice) (**a**). The percentage of Alizarin red-positive bone nodules area reported to the total well area was quantified with Image-J software. Results are represented as mean of 4 (n = 4) independent experiments, each performed in triplicates (**b**). Error bars correspond to SEM. Statistical analysis was performed using one-way ANOVA followed by Tukey’s multiple comparison test (*p < 0.05, **p < 0.005).

Another major bone cell type affected by GC excess is the osteoblast (OBL). We did not observe any *in vivo* effects on PINP serum levels and osteocalcin staining by histology (Fig. 2d–f) however assessing the direct effects on osteoblasts in isolation was investigated to determine whether these cells on their own are similarly less sensitive to GC like the OC (Fig. 4). In order to assess the GC osteogenic induction potential on C57BL/6 and CD1 OBLs, calvarial OBLs were isolated from 1 day old pups and cultured in control or osteogenic medium followed by identifying the mineralized calvarial bone nodules by alizarin red-S staining on day 6 of culture. Alizarin red-S staining revealed that the addition of phosphoascorbic acid and β-glycerolphosphate induced mineralization of calvarial OBLs in both mouse strains (Fig. 5a); however culturing OBLs only in control media did not result in mineralization (results not shown). The addition of Dex in different concentrations (0, 10^1, 10^2, 10^3 nM) to C57BL/6 mice OBLs did not significantly influence the bone nodule forming capacity of these cells, however a slight trend of dose response could be observed (higher doses of Dex seemed to induce more calcium deposition) (Fig. 5a,b). Bone nodule formation and mineralization in the case of CD1 OBLs was increased significantly by 10^2 nM Dex, while the high concentration of 10^3 nM inhibited OBL mineralization and bone nodule formation (Fig. 5a,b).

## Discussion

To date, corticosteroids are frequently used in the treatment of various chronic inflammatory diseases due to their potent anti-inflammatory immunosuppressive actions. However, corticosteroids are well known to cause osteopenia after long-term use and this side effect can cause major problems in clinical practice^1^,^18^. Several factors that facilitate GIO development have already been decoded^19^,^20^ but identification of still unknown regulators/inducers is presently on going with different transgenic and/or knockout mice.

As most genetically modified mice have been developed on C57BL/6 background we sought to compare the C57BL/6 strain propensity to develop GIO with CD1 mice that are regularly used as a well-established GIO experimental animal model. It is important to note that at baseline there is a significant difference in bone parameters, including maximum load, BV/TV, and cortical and trabecular thickness, between the CD1 and C57BL/6 mice. This has previously been reported and the C57BL/6 mice are known to have low skeletal mass compared to other mouse strains^21^. The CD1 mice are an outbred strain derived from the Swiss Webster mice and are known to have a significantly higher skeletal bone mass. As such it could be proposed that the C57BL/6 mice simply have less bone to lose and hence why GC treatment fails to obtain significant effect, however, this is an unlikely explanation as C57BL/6 mice are frequently used for ovariectomy-induced bone loss studies where a significant reduction in bone parameters is observed following estrogen withdrawl^22^.

The mechanical properties of bone are dependent on its composition (i.e. porosity and mineralization) and organisation (i.e. trabecular and cortical bone architecture, collagen fibre orientation); a decrease in bone mineral mass and density leads to a loss of bone stiffness and an increased fracture risk. As the first sign of osteoporosis is loss of bone strength and elasticity that often precede osteopenia we have evaluated these indices in the femur by using three-point bend testing. The three-point bend testing revealed that prednisolone treated CD1 femurs had reduced elasticity and tolerated less load compared to placebo treated mice whereas the biomechanical properties of C57BL/6 mice femurs were not affected by GC treatment.

Vertebral bone architectural parameters in prednisolone treated CD1 mice were characteristic of an osteoporotic trabecular structure with significantly reduced trabecular bone volume^11^ and trabecular number and increased trabecular pattern factor indicating lower trabecular bone connectivity. The structural model index was also increased in the GC treated CD1 mice indicating a transition of trabecular bone from plate-dominated to rod-dominated structures, a phenomenon often associated with bone fragility^23^. However, vertebral trabecular parameters in C57BL/6 mice in our experiments were not influenced by long term GC administration.

Histological analysis of the lumbar vertebrae after TRAP staining revealed that long term GC administration increased the number of OCL on the trabecular bone surface in case of the CD1 mice but did not affect significantly the C57BL/6 mice. As serum CTX levels act as marker of increased bone resorption due to OCL over activation in osteoporosis^24^, serum CTX was tested in prednisolone and placebo treated mice. Only CD1 mice presented increased serum CTX levels in response to long term CG treatment indicating accentuated bone resorption whilst C57BL/6 mice CTX levels were not affected.

In contrast to our *in vivo* findings, several studies do report successful induction of osteopenia in C57BL/6 mice after long term GC administration, but it is notable that in each case the mice were over 5 months of age at the start of the experiment and the GC induced bone loss was subtle^10^,^25^. We can just speculate that the mechanisms that prevent GIO induction in younger C57BL/6 mice may become apparent with advancement in age i.e. the increase in bone marrow fat at the expense of osteoblast formation that occurs with age. Furthermore, our result is partially in accordance with a study conducted by Weinstein, who found that in 6 month old C57BL/6 mice Cathepsin-K and Calcitonin receptor mRNA expression was not altered by prednisolone administration and *in situ* OC numbers were not affected either, but they observed a moderately reduced bone mineral density in the GC treated mice^26^.

As our *in vivo* data and several other studies suggest that OC development and activity is altered during long term GC exposure^27^,^28^,^29^,^30^,^31^ and knowing that OCs have an important role in the onset of the GIO^32^,^33^, we induced CD1 and C57BL/6 BM cells to from OCs *in vitro.* These studies revealed that OCs from CD1 mice displayed increased responsiveness to low doses of Dex and increased sensitivity to high doses of Dex. In the C57BL/6 mice low doses of Dex did not affect ostoclastogenic potential however the addition of high doses of Dex stimulated osteoclastogenesis and possibly also OC fusion or spreading as the OC size was increased as observed by visual inspection. Thus, our data obtained with C57BL/6 mice cells contradicts the literature where Dex was shown to have inhibitory effect, however the inhibitory effect of Dex on CD1 OCs is in agreement with previously published data^29^. It is worth mentioning that the study conducted by Kim *et al*., assessed only the C57BL/6 and not the CD1 BM cells response to different doses of Dex, and significant differences between the experimental conditions (e.g. OC purification) may have produced this inconsistency^29^. Nevertheless, in our hands CD1 mice OCs proved to be far more sensitive to the *in vitro* culture conditions regarding the Dex dose than the C57BL/6 cells. Furthermore, our data explains at least partially why the CD1 mice *in vivo* had increased OC numbers after GC treatment and showed increased propensity to develop GIO, but not the C57BL/6 mice.

Decreased OBL activity leading to reduced new bone formation due to long term GC administration is now considered to be one of the main causes of GIO^32^,^34^. Changes in OBL activity *in viv*o were not detected, most likely as these measurements were taken at the end stage of the disease. As previously reported, in our study phosphoascorbic acid and β-glycerolphosphate was sufficient *in vitro* to provide an inductive effect for OBL mineralization in both mouse strains^35^,^36^. Moreover, the ability of Dex at 10^2 nM dose to stimulate OBL mineralization is also in accordance with previous studies showing that GCs at physiological levels and GC signalling stimulate bone mineralization and bone development^37^,^38^. It is of importance to mention that the dose of 10^2 nM Dex represents the regular dose used for OBL induction media in most of the published recipes^38^,^39^. However, when the Dex dose was increased its detrimental effect became recognizable in the CD1 mice calvarial OBL cultures but not in the C57 BL/6 cell cultures.

Overall, CD1 mice responded as expected to long term GC treatment and it appears that prednisolone stimulated OCL maturation, activity and possibly life span^26^,^28^,^29^ leading to sustained bone loss^40^. As to why 12 weeks old C57BL/6 mice were not responsive to long term GC administration and eluded experimental GIO induction was revealed by *in vitro* studies. Both major bone cell types: OC and OBL from CD1 and C57BL/6 mice displayed different sensitivity levels towards different doses of Dex *in vitro*; CD1 cells were much more sensitive to reduced doses of GC as opposed to C57BL/6J bone cells that tolerated higher doses and seemed to be resistant to the deleterious effects of Dex administration. It is this inequality of OC and OBL sensitivity to different doses of GC in CD1 and C57BL/6J mice that we propose leads to the strain dependent differences in GIO susceptibility.

To our knowledge this is the first study that compares the CD1 and C57BL/6 strains susceptibility for the induction of experimental GIO in young animals (12 weeks) in depth and analyses the cellular components of bone that play critical role in GIO induction. Here we have demonstrated that GIO can be successfully established in the 12 weeks old CD1 mice but not in the C57BL/6 mice and infers that the genetic variability between these strains significantly contributes to the bone loss associated with long term GC administration. The differences reported here between the C57BL/6 and CD1 mice provide useful information that allows researchers to choose the appropriate genetic background for experimentally inducing GC related osteopenia and for the development of future transgenic and knockout mice involved in GIO research.

## Additional Information

**How to cite this article**: Ersek, A. *et al*. Strain dependent differences in glucocorticoid-induced bone loss between C57BL/6J and CD-1 mice. *Sci. Rep.* **6**, 36513; doi: 10.1038/srep36513 (2016).

**Publisher’s note:** Springer Nature remains neutral with regard to jurisdictional claims in published maps and institutional affiliations.
